# Psychological Well-Being in Clinical Research Coordinators

**DOI:** 10.1001/jamanetworkopen.2025.23985

**Published:** 2025-07-29

**Authors:** Regina M. Longley, M. Tim Song, Daniel A. Schaefer, Annabella C. Boardman, Emma P. Keane, Isabella S. Larizza, Emma D. Wolfe, Michelle Guo, Joseph Wu, Janet Abrahm, Hermioni L. Amonoo

**Affiliations:** 1 Icahn School of Medicine at Mount Sinai, New York, New York; 2 Department of Psychiatry, Massachusetts General Hospital, Boston; 3 Harvard Medical School, Boston, Massachusetts; 4 Department of Psychiatry, Brigham and Women’s Hospital, Boston, Massachusetts; 5 University of Washington School of Public Health, Department of Health Systems and Population Health, Seattle; 6 Department of Supportive Oncology, Dana-Farber Cancer Institute, Boston, Massachusetts

## Abstract

This cohort study examines demographic and psychological characteristics of clinical research coordinators to inform targeted workforce support strategies.

## Introduction

Clinical research coordinators (CRCs) are essential to the $93 billion^1^ US medical research enterprise, balancing administrative and participant-facing tasks to uphold research integrity.^2^ Little is known about CRCs’ demographics and psychological well-being despite their central role.^2,3^ High turnover, unclear role expectations, and limited training may contribute to psychological distress.^2^ Understanding CRC well-being is critical as burnout and job dissatisfaction could jeopardize data integrity and research continuity. As medical research flourishes, supporting CRCs is vital to sustaining a reliable research infrastructure. This study describes CRCs’ demographic and psychological characteristics to inform targeted workforce support strategies.

## Methods

This cross-sectional study was approved by the Mass General Brigham institutional review board and followed the STROBE reporting guideline. All participants provided informed consent electronically before completing the online surveys. We analyzed data collected from a cross-sectional survey of 358 primarily Massachusetts-based CRCs working (December 2023 to August 2024) with CRCs who have worked with human participants for 6 months or longer. Participants self-reported demographic and employment information and completed psychological scales for perceived social support, depression (measured by Center for Epidemiologic Studies Depression Scale [CES-D]), anxiety (measured by General Anxiety Disorder-7 [GAD-7]), and burnout (measured by Oldenburg Burnout Inventory [OLBI]) (eMethods in Supplement 1).

Analyses were conducted using RStudio 2024.09.0 Build 375 (Posit Software, PBC). Descriptive statistics were used to summarize participant characteristics and outcomes. Continuous psychological outcomes (GAD-7, CES-D, and total OLBI scores) were compared using independent sample *t* tests. Demographic variables with multiple levels were collapsed a priori into binary contrasts (eg, undergraduate degree or below vs graduate degree or above, man vs not man, heterosexual vs not heterosexual, single vs not single, living alone vs not living alone) due to small cell sizes and to preserve power. For each binary variable, the larger subgroup was the referent. We reported group mean (SD) and computed Cohen *d* for effect size (0.2, small; 0.5, medium; 0.8, large). Raw *P* values from *t* tests were adjusted for multiple comparisons using the Benjamini–Hochberg false-discovery-rate procedure, with 2-tailed α = .05.

## Results

Among 358 participants, most were from Massachusetts (90.2%), aged 35 years or younger (96.7%), female (85.8%), and heterosexual (70.9%); had household incomes of $25 000 to $49 999 (54.5%); and held 1 job (73.2%). Additionally, 44.1% were single, 31.6% in a relationship without cohabitation, and 77.4% held an undergraduate degree, primarily in Science, Technology, Engineering, and Mathematics (56.1%) (Table). CRCs worked in diverse disciplines, with median (IQR) work duration of 14.5 (11.0-24.0) months at time of survey completion.

**Table.** Baseline Demographic and Psychological Characteristics of Participants in the CRC Project

Characteristic	Participants, No. (%)
Total, No.	358
Age, y	
18-24	220 (61.5)
25-34	126 (35.2)
35-44	6 (1.7)
45-54	1 (0.3)
55-64	2 (0.6)
≥65	3 (0.8)
Gender	
Man	46 (12.8)
Woman	307 (85.8)
Queer, genderqueer, or nonbinary	5 (1.4)
Sexual orientation	
Asexual	1 (0.3)
Bisexual or pansexual	56 (15.6)
Gay, lesbian, or homosexual	18 (5.0)
Straight or heterosexual	254 (70.9)
Queer	19 (5.3)
Questioning or unsure	8 (2.2)
Other^a^	2 (0.6)
Ethnicity	
Hispanic or Latino/a/x	34 (9.5)
Not Hispanic or Latino/a/x	324 (90.5)
Race	
Asian, Asian American, or Asian background (including Indian subcontinent)	63 (17.6)
Black, African American, or African background	17 (4.7)
Middle Eastern	5 (1.4)
White or European background	250 (69.8)
More than 1 race	17 (4.7)
Other^b^	6 (1.7)
Relationship status	
In a relationship, not living together	113 (31.6)
Married or living with partner	86 (24.0)
Separated or divorced	1 (0.3)
Single	158 (44.1)
Education	
High school diploma or GED	1 (0.3)
Some college, associate’s degree, or technical or vocational school	3 (0.8)
College degree	277 (77.4)
Some postgraduate or professional education	20 (5.6)
Postgraduate, professional, or doctorate degree	57 (15.9)
Degree field	
Arts or humanities	17 (4.7)
Business	1 (0.3)
Health or medical studies	16 (4.5)
Interdisciplinary fields	60 (16.8)
Social sciences	63 (17.6)
STEM	201 (56.1)
Total annual household income	
>$25 000	5 (1.4)
$25 000-$49 999	195 (54.5)
$50 000-$74 999	83 (23.2)
$75 000-$99 999	18 (5.0)
$100 000-$149 999	22 (6.1)
≥$150 000	34 (9.5)
Missing	1 (0.3)
Second job	
No	262 (73.2)
Yes, in clinical research	10 (2.8)
Yes, not in clinical research	86 (24.0)
Living situation	
By myself	51 (14.2)
Family member(s)	37 (10.3)
Partner or spouse	72 (20.1)
Roommate(s) or friend(s)	181 (50.6)
Multiple living situations indicated (eg, with partner and roommates)	16 (4.5)
Other	1 (0.3)
Region of origin	
Midwest	35 (10.3)
East north central	26 (7.3)
West north central	9 (2.5)
Northeast	203 (59.9)
New England	147 (41.1)
Mid-Atlantic	56 (15.6)
South	44 (13.0)
South Atlantic	33 (9.2)
East south central	2 (0.6)
West south central	9 (2.5)
West	41 (12.1)
Mountain	7 (2.0)
Pacific	34 (9.5)
Puerto Rico	1 (0.3)
Outside of US	15 (4.2)
Missing	19 (5.3)
State of work	
MA	323 (90.2)
Not MA	16 (4.5)
Missing	19 (5.3)
Research focus	
Cardiovascular	24 (6.7)
Congenital disorders	3 (0.8)
Infectious diseases	5 (1.4)
Inflammatory and immune systems	8 (2.2)
Mental health, psychology, and/or psychiatry	62 (17.3)
Metabolic and endocrine disorders	11 (3.1)
Neurological	68 (19.0)
Oncology	44 (12.3)
Orthopedic	10 (2.8)
Pulmonary	7 (2.0)
Reproductive health	5 (1.4)
More than 1	56 (15.6)
Other	55 (15.4)
Duration of time worked as a CRC, median (IQR), mo	14.5 (11.0-24.0)
Psychological scale	
GAD-7 scale, mean (SD)	6.61 (5.29)
Minimal (0-4)	148 (41.3)
Mild (5-9)	105 (29.3)
Moderate (10-14)	54 (15.1)
Severe (15-21)	34 (9.5)
Missing	17 (4.7)
CES-D, mean (SD)	14.57 (9.90)
Minimal (0-9)	123 (34.3)
Mild (10-15)	82 (22.9)
Moderate (16-24)	77 (21.5)
Severe (25-60)	54 (15.1)
Missing	22 (6.1)
OLBI-Total, mean (SD)	41.28 (7.19)
OLBI-Disengagement subscale, mean (SD)	20.63 (4.01)
OLBI-Exhaustion subscale, mean (SD)	20.65 (4.19)
MSPSS-Total, mean (SD)	5.75 (0.97)
MSPSS-Family subscale, mean (SD)	5.55 (1.42)
MSPSS-Friends subscale, mean (SD)	5.86 (1.13)
MSPSS-Significant Others subscale, mean (SD)	5.85 (1.42)

Abbreviations: CES-D, Center for Epidemiologic Studies Depression Scale; CRC, clinical research coordinators; GAD-7, General Anxiety Disorder-7; GED, general educational development; MA, Massachusetts; MSPSS, Multidimensional Scale of Perceived Social Support; OLBI, Oldenburg Burnout Inventory; STEM, science, technology, engineering, mathematics.

^a^ Wrote in prefer not to answer or undefined.

^b^ Wrote in Brazilian, Jewish, Guyanese, Hispanic, North African.

CRCs reported high perceived social support (mean [SD], 5.75 [0.97]), particularly those in a relationship (d = 0.38; *P* = .001) and with graduate-level or greater education (d = 0.25; *P* = .04). GAD-7 scores (mean [SD], 6.61 [5.29]) indicated 24.6% had clinically significant anxiety. Men (d = 0.35; *P* = .02) and graduate degree or higher–bearing (d = 0.35; *P* = .006) participants reported lower anxiety scores, while nonheterosexual participants reported higher anxiety scores (d = 0.24; *P* = .048). CES-D scores (mean [SD], 14.57 [9.90]) indicated 36.6% had moderate-to-severe depression; nonheterosexual participants (d = 0.34; *P* = .004) and single participants reported higher depression scores (d = 0.24; *P* = .03). OLBI scores (mean [SD], 41.28 [7.19]) indicated higher burnout (d = 0.38; *P* = .04) and exhaustion (d = 0.44; *P* = .01) in those living alone (Figure).

**Figure.**
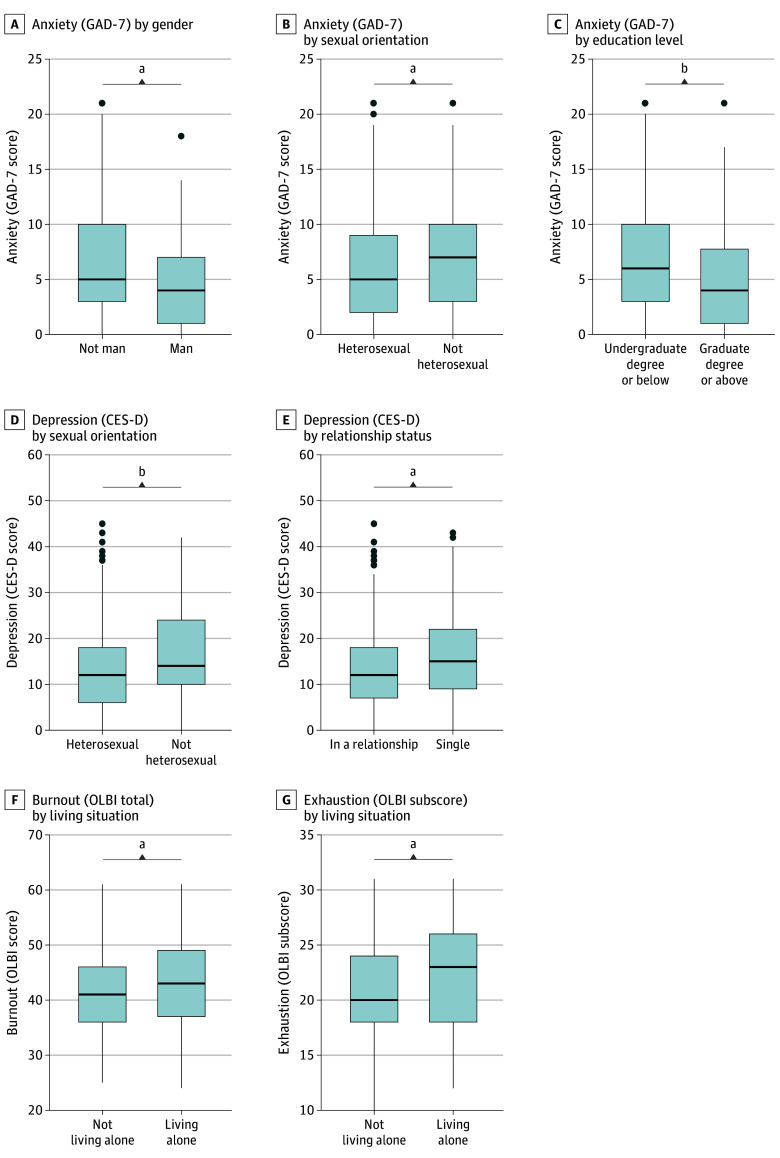
Significant Differences in Anxiety, Depression, and Burnout Scores by Demographic Group ^a^*P* < .05. ^b^*P* < .01. CES-D indicates Center for Epidemiologic Studies Depression Scale; GAD-7, General Anxiety Disorder-7; OLBI, Oldenburg Burnout Inventory.

## Discussion

This first comprehensive survey describing Massachusetts-based CRCs’ sociodemographic and psychological characteristics found most CRCs were young, female, heterosexual, not Hispanic, and White. Reported rates of clinically significant anxiety and depression exceeded prevalence within similar age groups,^4^ resembling elevated rates observed in medical students.^5^ Since many CRCs pursue medical school, compounding psychological risks may be concerning.

Role-specific stressors like heavy workload,^2^ role ambiguity, and career uncertainty may erode CRC well-being. Psychological distress varied across demographic groups, highlighting the need for targeted, contextualized support. Social support may buffer psychological distress, promotable by supportive work culture^3^ and peer support.^6^ The financial inaccessibility of low-paying CRC roles may also reinforce barriers to academia and medicine.^2^

This study’s cross-sectional design and homogeneous sample limit causal inference and generalizability. Our findings encourage further exploration of CRC well-being to uphold health outcomes and research advancements. Future research should examine role-specific stressors and sociodemographic underrepresentation. Institutions should consider peer support programs, wellness screenings, and structured interventions to promote CRC well-being.

## Notes

Supplement 1.
eMethods.

eReferences.

Supplement 2.
Data Sharing Statement
