# Supplementary material for: Psychological Well-Being in Clinical Research Coordinators
Source: JAMA Netw Open. 2025 Jul 29;8(7):e2523985. doi: 10.1001/jamanetworkopen.2025.23985 (PMC12308445; doi:10.1001/jamanetworkopen.2025.23985)
Supplement: Supplement 1. — eMethods. eReferences. [file jamanetwopen-e2523985-s001.pdf]

eMethodsSupplemental Online Content

Longley RM, Song MT, Schaefer DA, et al. Psychological well-being in Massachusetts-based clinical research coordinators. *JAMA Netw Open*. 2025;8(7):e2523985. doi:10.1001/jamanetworkopen.2025.23985

eMethods.
eReferences.

This supplemental material has been provided by the authors to give readers additional information about their work.

© 2025 Longley RM et al. *JAMA Network Open*.

eMethods.

This study was approved by the Mass General Brigham (MGB) Institutional Review Board. All participants provided informed consent electronically before completing the online surveys via the MGB Research Electronic Data Capture (REDCap) tools. As a cross-sectional study, this manuscript was developed in concordance with the STROBE reporting guidelines.

<u>Self-report measures</u>

Sociodemographic information

All sociodemographic information (race, ethnicity, gender, etc.) was self-reported by participants from categories pre-populated by the investigation team. As the main purpose of this study was to understand the demographic composition of clinical research coordinators, this information was necessary to collect. There were free text boxes available to write in responses if the participant selected the “Other” option.

Perceived social support

The 12-item Multidimensional Scale of Perceived Social Support (MSPSS)[1] was used to quantify perceived social support from family, friends, and significant others (range 1-7). Higher scores indicate stronger perceived social support. Specifically, scores from 1 to 2.99 are considered low; 3 to 5 moderate, and 5.1 to 7 high perceived social support.

Depression

The 20-item Center for Epidemiologic Studies Depression (CES-D)[2] scale was used to quantify depressive symptoms over a one-week period (range 0-60). Higher scores indicate

© 2025 Longley RM et al. *JAMA Network Open*.

greater severity of depressive symptoms, with scores from 0-9 indicating minimal, 10-15 mild, 16-24 moderate, and 25 or greater severe depressive symptoms. Scores above 16 indicate clinically significant depression.

Anxiety

The 7-item General Anxiety Disorder-7 (GAD-7)[3] was used to quantify anxiety symptoms over a two-week period (range 0-21). Higher scores indicate greater anxiety, with 0-4 indicating minimal, 5-9 mild, 10-14 moderate, and above 15 severe anxiety symptoms. Scores above 10 indicate clinically significant anxiety.

Burnout

The 16-item Oldenburg Burnout Inventory (OLBI)[4] was used to quantify occupational burnout and includes a disengagement and exhaustion subscale (subscale score range 8-32; total 16-64). Higher scores indicate greater levels of burnout.

Data analysis

Normality was assessed visually using histograms or the Shapiro-Wilk test. Homogeneity of variances was examined using Levene's test. For unequal variances, Welch's t-test was applied. Subgroup sizes were verified to ensure a minimum of 10 participants. Comparisons below this threshold were excluded. This analysis was exploratory in nature, with the aim of identifying potential associations for hypothesis generation. Although corrections for multiple comparisons were considered, no formal adjustments were applied to avoid excluding potentially meaningful results.

© 2025 Longley RM et al. *JAMA Network Open*.

Missing data

Of the total sample, 333 participants (93%) had complete data for the psychological measures. Participants with missing data (7%) were excluded from the analysis. Missingness was assumed to be at random; no imputation methods were applied.

© 2025 Longley RM et al. *JAMA Network Open*.

eReferences.

1. Zimet GD, Powell SS, Farley GK, Werkman S, Berkoff KA. Psychometric characteristics of the Multidimensional Scale of Perceived Social Support. *J Pers Assess*. Winter 1990;55(3-4):610-7. doi:10.1080/00223891.1990.9674095
2. Radloff LS. The CES-D Scale:A Self-Report Depression Scale for Research in the General Population. *Applied Psychological Measurement*. 1977;1(3):385-401. doi:10.1177/014662167700100306
3. Spitzer RL, Kroenke K, Williams JB, Löwe B. A brief measure for assessing generalized anxiety disorder: the GAD-7. *Arch Intern Med*. May 22 2006;166(10):1092-7. doi:10.1001/archinte.166.10.1092
4. Demerouti E, Bakker AB, Vardakou I, Kantas A. The convergent validity of two burnout instruments: A multitrait-multimethod analysis. *European journal of psychological assessment*. 2003;19(1):12.
5. Panjeh S, Nordahl-Hansen A, Cogo-Moreira H. Establishing new cutoffs for Cohen's d: An application using known effect sizes from trials for improving sleep quality on composite mental health. *Int J Methods Psychiatr Res*. Sep 2023;32(3):e1969. doi:10.1002/mpr.1969

© 2025 Longley RM et al. *JAMA Network Open*.
