# Supplementary material for: Psychological Well-Being in Clinical Research Coordinators
Source: JAMA Netw Open. 2025 Jul 29;8(7):e2523985. doi: 10.1001/jamanetworkopen.2025.23985 (PMC12308445; doi:10.1001/jamanetworkopen.2025.23985)
Supplement: Supplement 2. — Data Sharing Statement [file jamanetwopen-e2523985-s002.pdf]

Data Sharing Statement

Data
Data available: Yes
Data types: Deidentified participant data
How to access data: hermioni_amonoo@dfci.harvard.edu
When available: With publication

Supporting Documents
Document types: None

Additional Information
Who can access the data: Deidentified data will be made available upon request, to hermioni_amonoo@dfci.harvard.edu.
Types of analyses: Data will be made available for any purpose upon request.
Mechanisms of data availability: Data will be made available upon request to the principal investigator (hermioni_amonoo@dfci.harvard.edu) after approval of proposed data use and a signed data access agreement.
